# Performance of AI-based machine learning models for overall survival prediction in advanced hepatocellular carcinoma patients receiving immunoradiotherapy

**DOI:** 10.3389/fphar.2025.1719479

**Published:** 2025-11-20

**Authors:** Xiao Feng, Xiaonan Wang, Shengyuan Luo, Jianwei Zhou, Shanbao Ke

**Affiliations:** 1 Department of Oncology, Henan Provincial People’s Hospital, Zhengzhou University People’s Hospital, Zhengzhou, China; 2 Department of Endocrinology and Nephrology, Boxing People’s Hospital, Binzhou, Shandong, China; 3 Department of The Second Clinical Medical College, Changzhi Medical College, Changzhi, Shanxi, China

**Keywords:** artificial intelligence, machine learning model, hepatocellular carcinoma, immunoradiotherapy, targeted therapy

## Abstract

**Background:**

Hepatocellular carcinoma (HCC) remains a leading cause of cancer-related mortality worldwide. Although immunotherapy and targeted therapy have improved survival in advanced HCC, outcomes remain heterogeneous. Radiotherapy (RT) may enhance systemic treatment efficacy through local control and immunomodulation. Artificial intelligence (AI) offers opportunities to integrate multimodal data for individualized prognostic assessment.

**Methods:**

A total of 175 HCC patients were included in this study: 115 in the RT group (RT + immunotherapy + targeted therapy) and 60 in the non-RT group (immunotherapy + targeted therapy). Baseline characteristics were analyzed with chi-square and Mann–Whitney U tests. Overall survival (OS) was compared using the Kaplan–Meier method and log-rank test. Patients were randomly divided into a training cohort and a validation cohort (6:4 ratio). Prognostic factors were identified in the training cohort and incorporated into 101 machine learning (ML) algorithms. Model performance was assessed using the concordance index (C-index), receiver operating characteristic (ROC) curves, and risk score stratification.

**Results:**

The RT group achieved significantly longer OS than the non-RT group (median OS: 15.4 vs. 8.5 months, *P* = 0.003). Four variables (“Child,” “BCLC stage,” “Size,” and “Treatment”) were identified as prognostic factors. Among 101 ML models, the StepCox (forward) + Ridge model showed the best performance (C-index: 0.68 in training, 0.65 in validation). Time-dependent ROC analysis demonstrated AUC values of 0.72, 0.75, and 0.74 at 1-, 2-, and 3-year OS in the training cohort, and 0.72, 0.75, and 0.73 in the validation cohort, respectively.

**Conclusion:**

RT significantly improved prognosis in advanced HCC patients treated with immunotherapy and targeted therapy. Among multiple algorithms, the StepCox (forward) + Ridge model achieved superior predictive performance, supporting its potential value in individualized prognostic assessment.

## Introduction

### Background

Hepatocellular carcinoma (HCC) is a major global health burden and represents the predominant histological subtype of primary liver cancer. It ranks as the sixth most frequently diagnosed malignancy and the third leading cause of cancer-related death worldwide, accounting for nearly 900,000 new cases and over 800,000 deaths each year (Moris et al., 2025). The incidence is particularly high in East Asia and sub-Saharan Africa, largely attributable to chronic hepatitis B and C infection, although alcohol consumption and the increasing prevalence of metabolic liver disease are emerging contributors (Yeo et al., 2025). Despite advances in surveillance strategies and curative interventions, the majority of patients are diagnosed at an advanced stage, where radical resection or transplantation is rarely feasible (Zhao et al., 2025; Chen et al., 2024; Melero et al., 2024). For these patients, systemic and locoregional approaches remain the cornerstone of treatment.

In the last decade, immunotherapy and targeted agents have substantially reshaped the treatment paradigm for advanced HCC (Jiang et al., 2024). Immune checkpoint inhibitors (ICIs), especially when combined with anti-angiogenic therapies such as bevacizumab, have demonstrated superior survival outcomes compared to sorafenib in phase III clinical trials, establishing immunotherapy-based combinations as standard first-line options (Zhao et al., 2025; Toh et al., 2022-02). In addition, tyrosine kinase inhibitors (TKIs) including lenvatinib, regorafenib, and cabozantinib continue to play a critical role in sequential treatment (Wang et al., 2023; Deng et al., 2025; Ahn et al., 2025). Nevertheless, therapeutic efficacy remains heterogeneous; primary resistance, acquired resistance, and immune-related adverse events limit durable benefit, underscoring the urgent need for predictive tools and individualized treatment strategies (Maas et al., 2025).

The role of radiotherapy (RT) in HCC has been redefined with the advent of modern high-precision techniques such as stereotactic body radiotherapy (SBRT) and intensity-modulated radiotherapy (IMRT) (Chen et al., 2022; Zhao et al., 2024). RT not only achieves meaningful local tumor control, especially in cases with vascular invasion or unresectable disease, but also exerts profound immunomodulatory effects. Preclinical and clinical studies suggest that RT enhances tumor antigen release, augments dendritic cell priming, and facilitates systemic immune activation, thereby providing a biological rationale for synergy with ICIs. This dual function renders RT a compelling component of multimodal regimens for advanced HCC (Cui et al., 2025; Cortese et al., 2025).

Artificial intelligence (AI) technologies have emerged as powerful tools for precision oncology (Rajak et al., 2025). In HCC, AI-driven radiomics, machine learning, and deep learning models have been employed to predict survival, recurrence, treatment response, and biomarker expression. By integrating clinical, imaging, and molecular data, AI can uncover complex nonlinear relationships beyond the scope of conventional statistics. Such approaches offer opportunities to improve patient stratification and support decision-making in the increasingly complex therapeutic landscape of advanced HCC (Teng et al., 2025).

Considering the limitations of current prognostic markers and the heterogeneity of clinical outcomes, we designed this study to establish and validate an AI-based prognostic model for patients undergoing immunotherapy, targeted therapy, and radiotherapy. By leveraging multimodal data integration, we aimed to refine risk assessment, identify subgroups with distinct survival trajectories, and ultimately provide a framework for personalized management in advanced HCC.

## Methods

### Patients

A total of 175 patients with HCC were enrolled from three tertiary hospitals. Among them, 60 patients received combined immunotherapy and targeted therapy (non-RT group), while 115 patients underwent radiotherapy in addition to immunotherapy and targeted therapy (RT group).

Inclusion criteria were as follows: (1) HCC confirmed by imaging or histopathology; (2) Barcelona Clinic Liver Cancer (BCLC) stage B or C; (3) Child–Pugh class A or B liver function; and (4) complete clinical data available.

Exclusion criteria included: (1) concomitant malignancies other than HCC; (2) patients unsuitable for radiotherapy; (3) presence of hepatic encephalopathy or refractory malignant ascites; and (4) loss to follow-up.

Because this study was retrospective in nature and all patient data were anonymized and unidentifiable, ethical approval was not required from the Ethics Committee of Henan Provincial People’s Hospital. The study was conducted in accordance with the ethical standards of the Declaration of Helsinki.

### Treatment and endpoints

All patients received combined targeted therapy and immunotherapy as the systemic treatment backbone. Patients who additionally underwent radiotherapy were classified into the RT group, while those who did not receive radiotherapy were classified into the non-RT group. The decision to administer radiotherapy was made by a multidisciplinary tumor board at each participating center, taking into account tumor burden, vascular invasion, liver function, performance status, and patient preference. Radiotherapy was delivered in accordance with institutional protocols, with individualized treatment planning to ensure both tumor control and normal liver sparing.

The primary endpoint of this study was overall survival (OS), defined as the time from initiation of treatment to death from any cause or the last follow-up.

### Statistical analysis

Categorical variables were compared using the chi-square test, while continuous variables were analyzed with the Mann–Whitney U test. OS between the RT and non-RT groups was estimated using the Kaplan–Meier (KM) method and compared with the log-rank test. To minimize potential selection bias and balance baseline characteristics between the two groups, propensity score matching (PSM) was performed using a 1:1 nearest-neighbor matching without replacement approach. The propensity score was calculated based on the following covariates: sex, age, Child–Pugh class, AFP level, BCLC stage, tumor number, tumor size, presence of portal vein tumor thrombosis (PVTT), lymph node involvement (N), and extrahepatic metastasis (M). All patients were randomly divided into a training cohort and a validation cohort at a 6:4 ratio. Univariate Cox regression was performed to identify prognostic factors associated with OS. Clinical variables included sex, age, Child–Pugh class, AFP, BCLC stage, tumor number, tumor size, PVTT, N, and M. Variables with p < 0.05 were selected for subsequent machine learning (ML) modeling. The predictive performance of each model was comprehensively assessed using the concordance index (C-index), receiver operating characteristic (ROC) curves, and risk score stratification. Internal validation was performed using the pre-defined validation cohort to ensure model robustness.

## Result

### Patients

A total of 175 patients with HCC were included in the analysis. The overall cohort was predominantly male (85.1%), with a mean age of 52.5 years. Chronic hepatitis B virus (HBV) infection was present in 70.3% of patients. Most patients had preserved liver function (Child–Pugh A, 76.6%) and advanced disease (BCLC stage C, 85.7%). Elevated serum AFP levels (≥400 ng/mL) were observed in 57.1% of patients. The majority had multifocal disease (85.7%), tumors ≥3 cm in diameter (78.9%), PVTT (58.9%), nodal involvement (57.7%), and extrahepatic metastasis (34.9%).

Before PSM, when comparing baseline characteristics between the RT group (n = 115) and the non-RT group (n = 60), no significant differences were found in sex distribution, age, HBV status, Child–Pugh class, BCLC stage, tumor number, tumor size, PVTT, nodal status, or metastatic disease (all P > 0.05). However, patients in the RT group had a higher proportion of AFP ≥400 ng/mL compared with the non-RT group (63.5% vs. 45.0%, P = 0.029).

After performing propensity score matching (PSM) with a 1:1 nearest-neighbor approach, 57 matched pairs were obtained. Following matching, no significant differences were observed between the RT and non-RT groups across all baseline variables (all P > 0.05), indicating that the two cohorts were well balanced (Table 1).

**TABLE 1:** Clinical features stratified by RT and Non-RT before and after PSM.

Characteristic		Before PSM			After PSM		P
	All	Non-RT	RT	P	Non-RT	RT	
	N = 175	N = 60	N = 115		N = 57	N = 57	
Sex				0.478			1.000
Female	26 (14.9%)	11 (18.3%)	15 (13.0%)		10 (17.5)	9 (15.8)	
Male	149 (85.1%)	49 (81.7%)	100 (87.0%)		47 (82.5)	48 (84.2)	
Age, mean ± SD	52.5 (11.0)	53.2 (12.1)	52.2 (10.4)	0.571	53.39 (12.28)	52.26 (10.34)	0.599
HBV				0.352			0.223
No	52 (29.7%)	21 (35.0%)	31 (27.0%)		21 (36.8)	14 (24.6)	
Yes	123 (70.3%)	39 (65.0%)	84 (73.0%)		36 (63.2)	43 (75.4)	
Child				0.868			0.823
A	134 (76.6%)	45 (75.0%)	89 (77.4%)		43 (75.4)	45 (78.9)	
B	41 (23.4%)	15 (25.0%)	26 (22.6%)		14 (24.6)	12 (21.1)	
AFP				0.029			0.574
<400	75 (42.9%)	33 (55.0%)	42 (36.5%)		30 (52.6)	26 (45.6)	
≥400	100 (57.1%)	27 (45.0%)	73 (63.5%)		27 (47.4)	31 (54.4)	
BCLC				0.626			0.599
B	25 (14.3%)	7 (11.7%)	18 (15.7%)		7 (12.3)	10 (17.5)	
C	150 (85.7%)	53 (88.3%)	97 (84.3%)		50 (87.7)	47 (82.5)	
Number				0.380			0.787
1	25 (14.3%)	11 (18.3%)	14 (12.2%)		9 (15.8)	7 (12.3)	
≥2	150 (85.7%)	49 (81.7%)	101 (87.8%)		48 (84.2)	50 (87.7)	
Size				0.317			0.881
<3	37 (21.1%)	13 (21.7%)	24 (20.9%)		12 (21.1)	14 (24.6)	
≥3,<5	64 (36.6%)	26 (43.3%)	38 (33.0%)		24 (42.1)	24 (42.1)	
≥5,<10	74 (42.3%)	21 (35.0%)	53 (46.1%)		21 (36.8)	19 (33.3)	
PVTT				1.000			0.847
No	72 (41.1%)	25 (41.7%)	47 (40.9%)		23 (40.4)	21 (36.8)	
Yes	103 (58.9%)	35 (58.3%)	68 (59.1%)		34 (59.6)	36 (63.2)	
N				0.313			1.000
No	74 (42.3%)	29 (48.3%)	45 (39.1%)		26 (45.6)	25 (43.9)	
Yes	101 (57.7%)	31 (51.7%)	70 (60.9%)		31 (54.4)	32 (56.1)	
M				0.062			
No	114 (65.1%)	33 (55.0%)	81 (70.4%)		33 (57.9)	33 (57.9)	1.000
Yes	61 (34.9%)	27 (45.0%)	34 (29.6%)		24 (42.1)	24 (42.1)	

### OS

**FIGURE 1:**
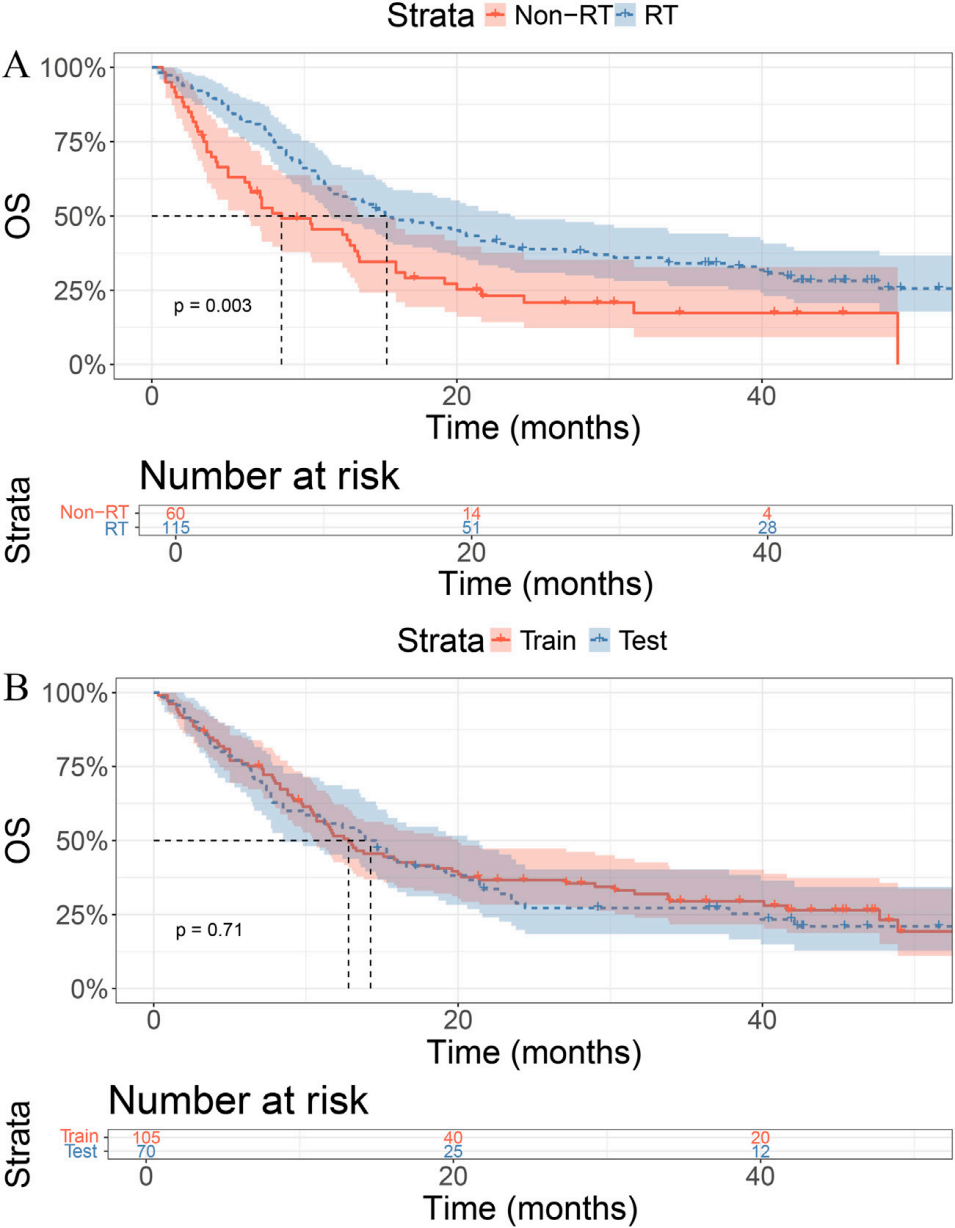
Kaplan–Meier curves for overall survival (OS). **(A)** Patients receiving RT showed better OS than those without RT (p = 0.003). **(B)** No significant OS difference between training and test sets (p = 0.71).

Before PSM, the median OS in the RT group was 15.4 months (95% CI: 11.7–23.5), while that in the non-RT group was 8.5 months (95% CI: 6.4–13.6), showing a statistically significant difference (P = 0.003; Figure 1A). After PSM, the median OS was 17.1 months (95% CI: 12.4–40.1) in the RT group and 8.5 months (95% CI: 6.1–13.6) in the non-RT group, with a statistically significant difference remaining (P = 0.0054; Supplementary Figure S1).

In the training cohort, the median OS was 14.2 months in the RT group and 12.8 months in the control group, with no statistically significant difference (P = 0.71, Figure 1B).

### Model construction

**FIGURE 2:**
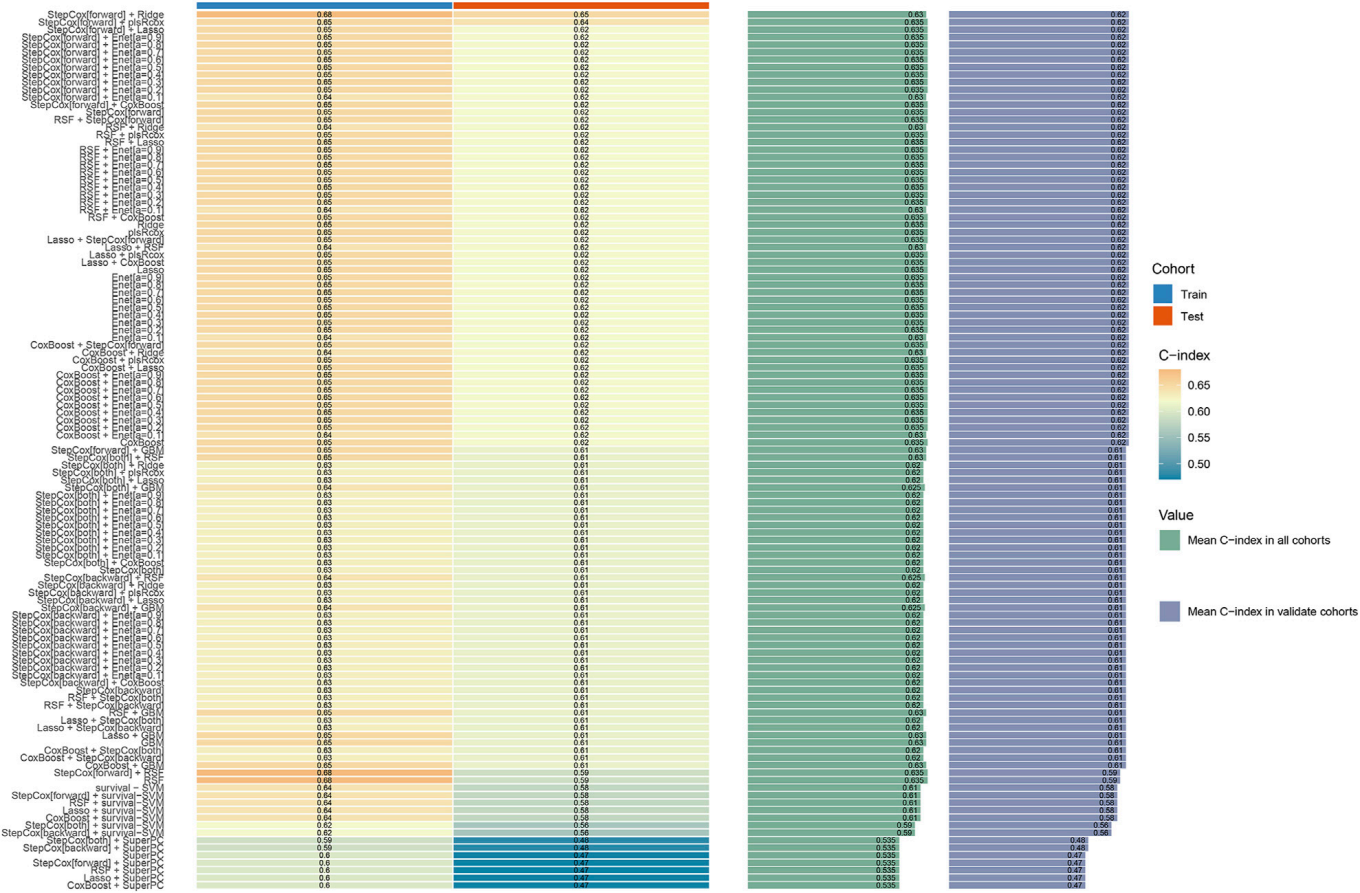
The concordance index (C-index) of 101 machine learning models in the training and validation cohorts.

**FIGURE 3:**
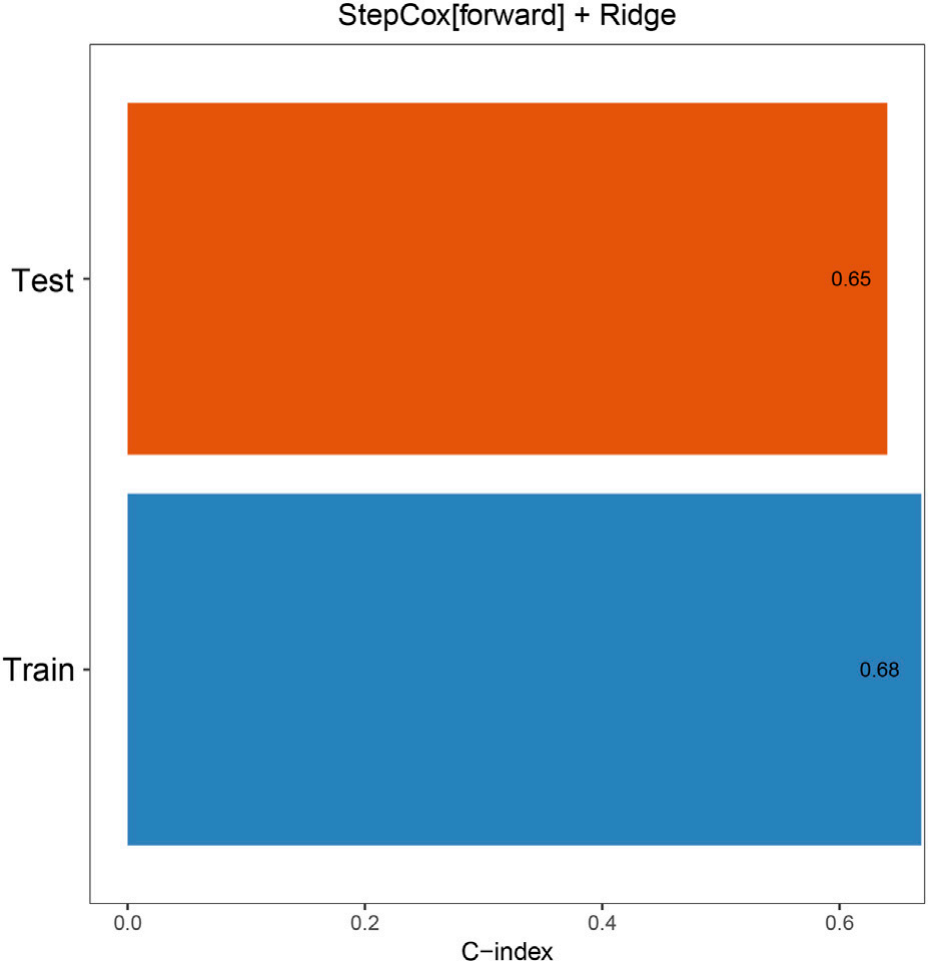
The concordance index (C-index) of StepCox (forward) + Ridge model in the training and validation cohorts.

There were no significant differences in baseline characteristics between the training and validation cohorts (all P > 0.05, Table 2). In the training cohort, univariate T-test was first used to identify four risk factors: “Child”, “BCLC”, “Size”, and “Treatment”. These factors were subsequently incorporated into 101 machine learning models. Among them, the StepCox (forward) + Ridge model demonstrated the highest C-index (0.68 in the training cohort and 0.65 in the validation cohort, Figures 2, 3).

**TABLE 2:** Clinical features stratified by Train and Test cohorts.

Characteristic	All	Train	Test	P
	N = 175	N = 105	N = 70	
Sex	26 (14.9%)	16 (15.2%)	10 (14.3%)	
Female	149 (85.1%)	89 (84.8%)	60 (85.7%)	
Male	52.5 (11.0)	52.5 (9.58)	52.6 (12.9)	0.962
Age, mean ± SD				0.661
HBV	52 (29.7%)	33 (31.4%)	19 (27.1%)	
No	123 (70.3%)	72 (68.6%)	51 (72.9%)	
Yes				0.086
Child				1
A	134 (76.6%)	80 (76.2%)	54 (77.1%)	
B	41 (23.4%)	25 (23.8%)	16 (22.9%)	
AFP	75 (42.9%)	51 (48.6%)	24 (34.3%)	
<400	100 (57.1%)	54 (51.4%)	46 (65.7%)	
≥400				0.123
BCLC	25 (14.3%)	19 (18.1%)	6 (8.57%)	
B	150 (85.7%)	86 (81.9%)	64 (91.4%)	
C				1
Number	25 (14.3%)	15 (14.3%)	10 (14.3%)	
1	150 (85.7%)	90 (85.7%)	60 (85.7%)	
≥2				0.303
Size	37 (21.1%)	26 (24.8%)	11 (15.7%)	
<3	64 (36.6%)	35 (33.3%)	29 (41.4%)	
≥3,<5	74 (42.3%)	44 (41.9%)	30 (42.9%)	
≥5,<10				0.301
PVTT	72 (41.1%)	47 (44.8%)	25 (35.7%)	
No	103 (58.9%)	58 (55.2%)	45 (64.3%)	
Yes				0.731
N	74 (42.3%)	46 (43.8%)	28 (40.0%)	
No	101 (57.7%)	59 (56.2%)	42 (60.0%)	
Yes				0.496
M	114 (65.1%)	71 (67.6%)	43 (61.4%)	
No	61 (34.9%)	34 (32.4%)	27 (38.6%)	

**FIGURE 4:**
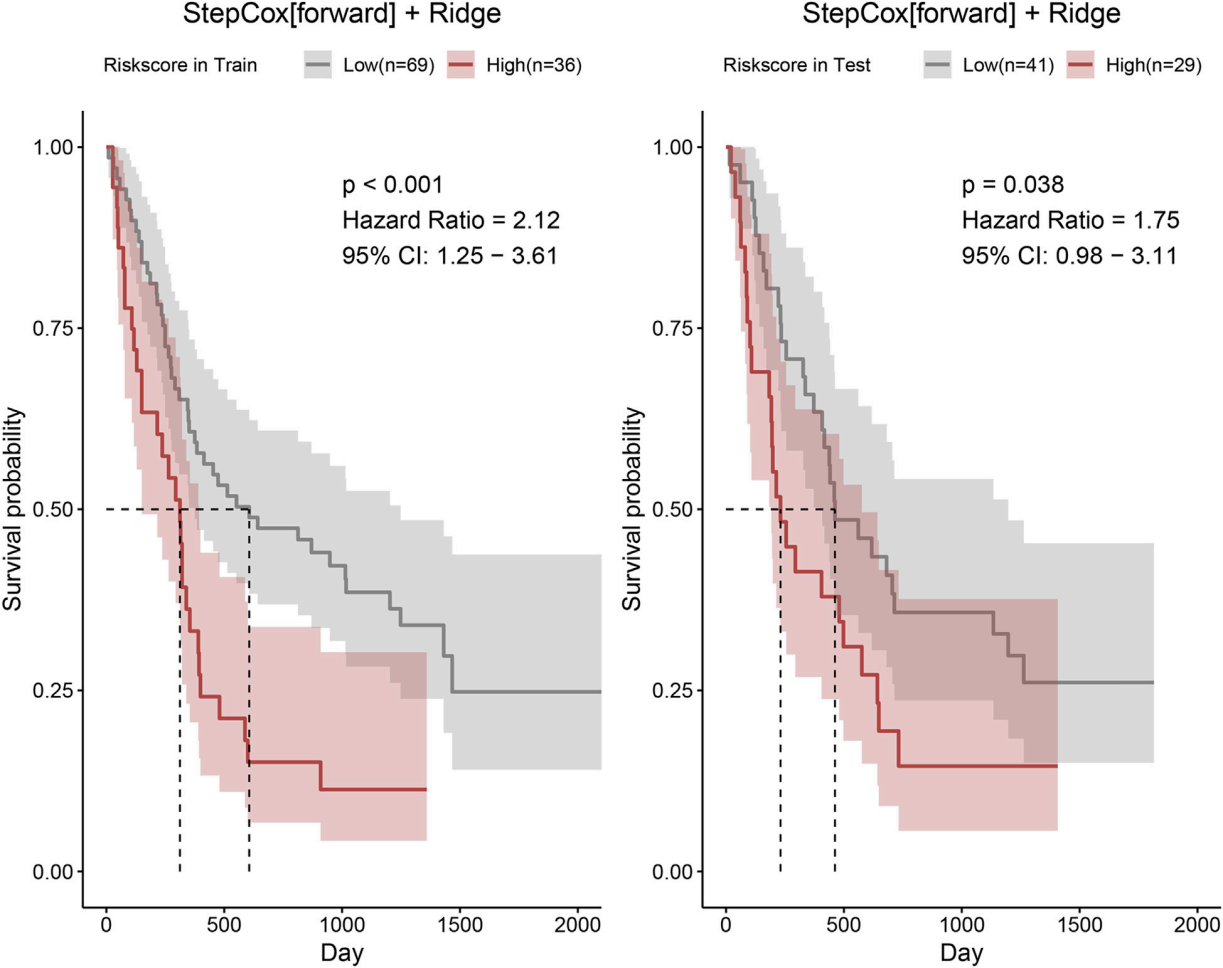
Risk scores of patients in the training and validation cohorts were calculated using the StepCox (forward) + Ridge model. Patients were then stratified into high- and low-risk groups according to the median risk score, and Kaplan–Meier survival curves were plotted.

Thereafter, risk scores for all patients were calculated using the StepCox (forward) + Ridge model, and the median value was used as the cutoff to stratify patients into high-risk and low-risk groups. The two risk groups showed good discriminative performance in both the training and validation cohorts (Figure 4).

**FIGURE 5:**
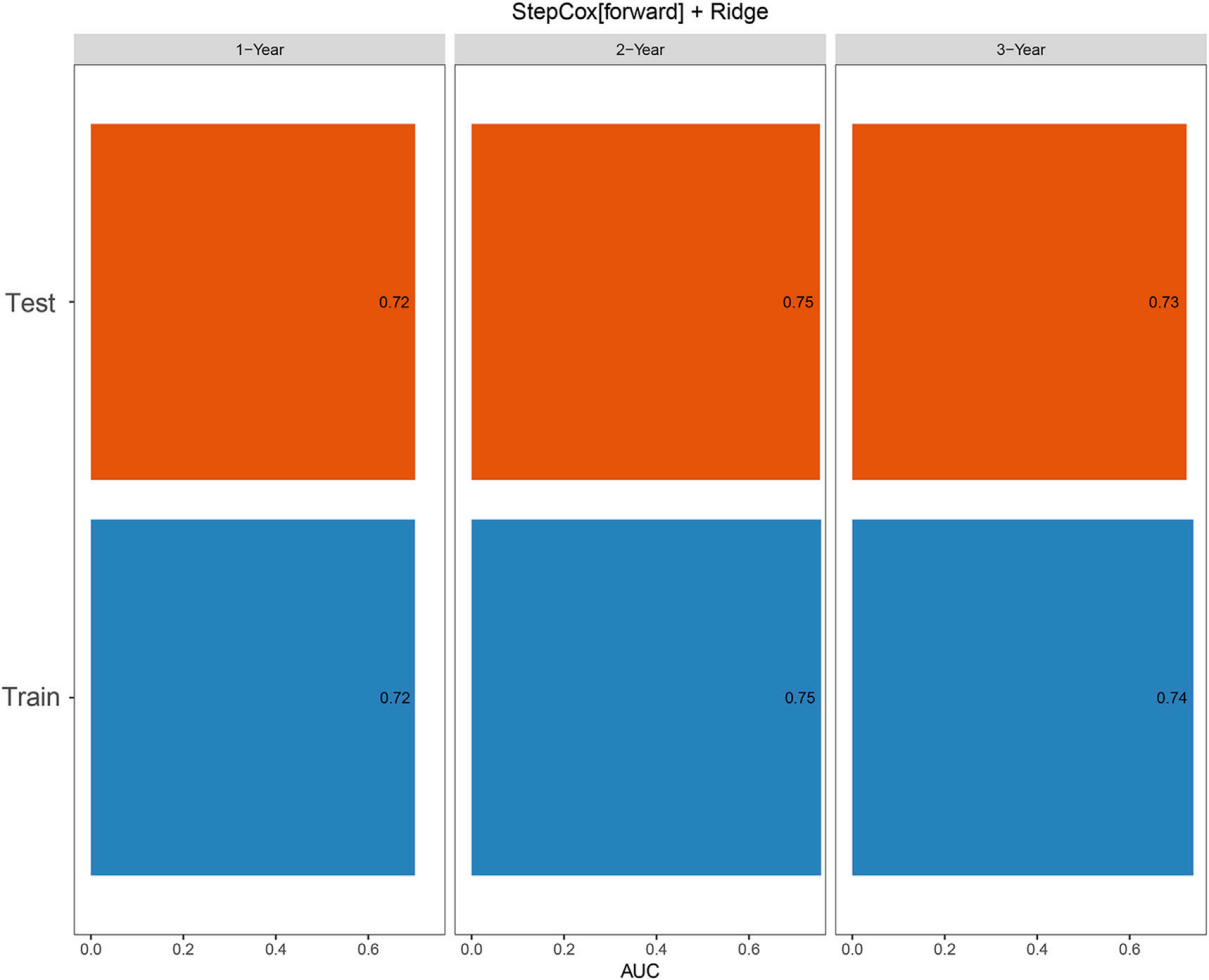
Time-dependent ROC curves for predicting 1-, 2-, and 3-year overall survival (OS) in the training and validation cohorts.

The time-dependent ROC analysis for predicting 1-, 2-, and 3-year OS yielded AUC values of 0.72, 0.75, and 0.74 in the training cohort, and 0.72, 0.75, and 0.73 in the validation cohort, respectively (Figure 5).

## Discussion

In this study, we demonstrated that the addition of RT to immunotherapy and targeted therapy significantly improved overall survival in patients with advanced HCC. Using multicenter clinical data, RT was shown to provide a meaningful survival advantage even after PSM adjustment, highlighting its potential role in enhancing systemic treatment efficacy.

Building upon these clinical findings, we developed and validated an AI-based prognostic model to further refine individualized risk assessment in this population. By integrating clinical and treatment-related variables, we systematically compared multiple machine learning algorithms and identified the StepCox (forward) + Ridge model as the optimal framework. This model exhibited robust discrimination and calibration across both the training and validation cohorts, supporting the feasibility of applying AI-driven approaches to guide prognostic evaluation in multimodal treatment settings.

Our findings also reinforce the clinical relevance of RT in patients undergoing immunotherapy plus targeted therapy. In the overall cohort, patients who received RT achieved significantly longer overall survival compared with those who did not. This survival benefit is consistent with a growing body of evidence suggesting that RT not only improves local tumor control but also acts as an immune modulator (Xu et al., 2025). Mechanistically, RT promotes tumor antigen release, enhances antigen-presenting cell priming, and increases effector T-cell infiltration into the tumor microenvironment. Moreover, RT may counteract immunotherapy resistance by reshaping the immunosuppressive milieu, for example, through modulation of myeloid-derived suppressor cells or regulatory T cells (Uribe-Herranz et al., 2020; Li et al., 2022; Kumari et al., 2020). These synergistic effects provide a compelling biological rationale for combining RT with systemic therapies in advanced HCC.

The integration of AI into oncology research opens new opportunities for precision medicine in HCC (Bo et al., 2024). Traditional statistical models are often limited in their ability to handle high-dimensional and heterogeneous data, while AI-based approaches can capture nonlinear interactions among clinical, imaging, and molecular variables. In this study, AI enabled us to screen numerous machine learning algorithms and objectively identify the best-performing model. As radiomics, genomics, and immunological biomarkers become increasingly available, future AI models are expected to integrate these multimodal data sources, thereby enhancing predictive accuracy and enabling individualized treatment selection (Zeng et al., 2023; Wang et al., 2024).

The StepCox (forward) + Ridge model demonstrated superior performance compared with conventional Cox regression and other ML algorithms tested in this study (Li et al., 2025; Su et al., 2025). The StepCox (forward) procedure enhances variable selection efficiency by iteratively including variables with the strongest prognostic contribution, which improves interpretability and reduces model complexity (Chen et al., 2025). In contrast, the Ridge component applies L2 regularization to mitigate multicollinearity and prevent overfitting, thereby increasing model stability and robustness across datasets (Yıldırım and Özkale, 2023). By combining these two complementary strengths, the StepCox (forward) + Ridge framework achieves a balance between interpretability and predictive accuracy, making it particularly suitable for clinical prognostic modeling in heterogeneous populations such as advanced HCC.

Among the variables included in our model, “Child”, “BCLC stage”, “tumor size”, and “treatment modality” emerged as significant prognostic factors. These findings are in line with the clinical understanding of HCC biology. Child–Pugh classification reflects the underlying hepatic functional reserve, which is crucial in determining treatment tolerance and long-term survival (Melinder et al., 2023). BCLC staging, widely adopted in clinical practice, stratifies patients by tumor burden, liver function, and performance status, providing an established prognostic framework (Trevisani et al., 2024). Tumor size remains an important indicator of disease aggressiveness and metastatic potential (Shehta et al., 2024). Finally, treatment modality, particularly the addition of RT, directly influences survival outcomes, as demonstrated in our analysis. Together, these factors not only strengthen the validity of the model but also reflect the biological and clinical heterogeneity of HCC.

From a clinical perspective, the proposed AI-based StepCox (forward) + Ridge prognostic model offers substantial value in the management of advanced HCC. By integrating readily available clinical and treatment-related parameters, the model enables clinicians to identify patients who are most likely to derive meaningful benefit from the addition of RT to immunotherapy and targeted therapy. Through individualized risk stratification, patients can be categorized into high- and low-risk groups, allowing clinicians to better tailor treatment plans and patient counseling (Wang et al., 2025; Zhang et al., 2024).

Low-risk patients may be expected to achieve durable survival benefits and could continue standard RT-based multimodal therapy, while high-risk patients may warrant closer monitoring, early treatment intensification, or enrollment in clinical trials exploring novel therapeutic strategies. Furthermore, this AI-driven model can serve as a decision-support tool in multidisciplinary tumor boards, complementing existing clinical guidelines and promoting evidence-based, personalized management of advanced HCC.

Nevertheless, several limitations of this study should be acknowledged. First, the analysis was retrospective in design, and although multicenter data were included, inherent biases such as patient selection and treatment heterogeneity cannot be fully excluded. Second, the sample size, particularly of the non-RT group, was relatively modest, which may limit the generalizability of the findings. Third, although the model demonstrated stable predictive performance in internal validation, external validation using independent, multi-institutional cohorts was not performed, and its generalizability requires further confirmation in prospective studies. Finally, although multiple machine learning algorithms were evaluated, the incorporation of imaging or molecular biomarkers was not feasible in the present dataset; future work integrating radiomics and omics-based features may further enhance model accuracy and clinical applicability.

In summary, this study presents a novel AI-based prognostic model for advanced HCC patients treated with RT in combination with immunotherapy and targeted therapy. The model demonstrated robust performance, identified clinically meaningful prognostic factors, and provided a framework for individualized risk stratification.

## Data Availability

All data generated or analyzed during this study are included in this article. Further enquiries can be directed to the corresponding author (X18716189260@163.com).
