# Supplementary material for: Performance of AI-based machine learning models for overall survival prediction in advanced hepatocellular carcinoma patients receiving immunoradiotherapy
Source: Front Pharmacol. 2025 Nov 20;16:1719479. doi: 10.3389/fphar.2025.1719479 (PMC12675426; doi:10.3389/fphar.2025.1719479)

Supplementary Fig.1 Kaplan-Meier curves for overall survival (OS) in the RT and Non-RT groups. The RT group showed a significantly better survival (p = 0.0054).

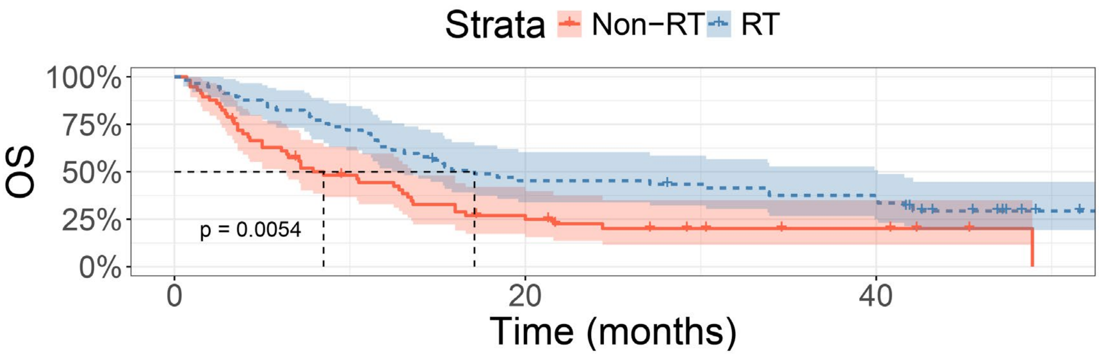

Supplement: Supplementary file 1 [file Image1.pdf]
